# Hemoglobin level is negatively associated with sarcopenia and its components in Chinese aged 60 and above

**DOI:** 10.3389/fpubh.2023.1081843

**Published:** 2023-03-13

**Authors:** Qiaoling Liu, Jiuhong You, Min Zhong, Zhigang Wu, Yunjie Geng, Cheng Huang

**Affiliations:** ^1^School of Cardiovascular and Metabolic Health, University of Glasgow, Glasgow, United Kingdom; ^2^Department of Rehabilitation Medicine, West China Hospital, Sichuan University, Chengdu, Sichuan, China; ^3^Department of Geriatric Neurology, Affiliated Brain Hospital of Nanjing Medical University, Nanjing, Jiangsu, China; ^4^Department of Chemistry, Southern University of Science and Technology, Shenzhen, Guangdong, China; ^5^Research Institute of Statistical Sciences, National Bureau of Statistics, Beijing, China; ^6^Key Laboratory of Rehabilitation Medicine in Sichuan Province, West China Hospital, Sichuan University, Chengdu, Sichuan, China

**Keywords:** China, adult, aging, hemoglobin, sarcopenia, epidemiology

## Abstract

**Introduction:**

Sarcopenia and low hemoglobin level are common in older adults. Few studies have evaluated the association between hemoglobin level and sarcopenia and with inconsistent findings. The multifaceted effects of sarcopenia on the human body and the high prevalence of anemia in the Chinese population make it necessary to explore the association between the two.

**Methods:**

Using the China Health and Retirement Longitudinal Study (CHARLS), we explored the association between hemoglobin with sarcopenia and its components in the Chinese population aged 60 and above. Multivariate logistic and Cox proportional hazards models were constructed to examine the association of hemoglobin level with sarcopenia and sarcopenia components in individuals aged 60 years or above. The subgroup analysis covered residence, body mass index level, drinking status, and smoking status were conducted. The possible difference of associations between sexes was also explored.

**Results:**

With a total of 3,055 people, the hemoglobin concentration in people without sarcopenia, possible sarcopenia, and sarcopenia are 14.34 ± 2.22, 14.64 ± 2.27, and 13.58 ± 2.02 g/dl, respectively. Cross-sectional analysis showed strong evidence that hemoglobin was negatively associated with sarcopenia [Odds Ratio (OR) = 0.95, 95% Confidence Interval (CI): 0.90–0.99] and low height-adjusted appendicular skeletal muscle mass (OR = 0.91, 95% CI: 0.86–0.97). On average, a per 1 g/dl higher hemoglobin level was associated with 5% lower odds of sarcopenia (OR = 0.95, 95% CI: 0.90–0.98). The cohort study of 1,022 people demonstrated a statistically significant negative association of hemoglobin level with low physical performance [Hazard Ratio (HR) = 0.92, 95% CI: 0.85–0.99], merely with sarcopenia (HR = 0.92, 95% CI: 0.84–1.00) and skeletal muscle mass (HR = 0.95, 95% CI: 0.80–1.00). Sex-specific analysis suggested hemoglobin's association with sarcopenia, muscle mass, and physical performance in all sexes, with weaker magnitudes in females. Hemoglobin in urban residents and people with high body mass index (BMI) has a larger magnitude of the negative association with sarcopenia.

**Discussion:**

Hemoglobin level associates with sarcopenia, muscle mass, and physical performance in the Chinese population aged 60 and above, with sex-specific, residence-specific, and BMI-specific effects.

## Introduction

Sarcopenia is a syndrome of progressive loss of muscle mass, strength, and physiological function of the muscles as people age. It associates with mortality and a decline in physical function. The physical and functional decline associated with sarcopenia can have serious negative impacts on an individual's quality of life. People suffering from sarcopenia often experience reduced independence, which can lead to feelings of isolation and depression (1). Additionally, they are more likely to suffer from chronic illnesses, such as type 2 diabetes mellitus and heart failure, which can further reduce their quality of life (2, 3).

The prevalence of sarcopenia in older Asian individuals ranges between 2.5 and 45.7% (4). In China, this number is between 8.9 and 38.8% (5). The seventh national population census in 2020 showed that the proportion of people aged 60 years and above in China is 18.7%, a total of 260 million people (6). Risk factors related to sarcopenia, such as obesity and diabetes mellitus, have an upward trend (7, 8). These findings suggested that China has a large vulnerable population, together with a high prevalence of sarcopenia. The lack of awareness of sarcopenia in clinical practitioners further casts shadows on healthy aging (9).

Anemia is a recognized risk factor for fatigue, mortality, and decreased functional capacity in elder individuals (10). The Chinese population has a high prevalence of anemia with considerable geographic differences. In middle and eastern China, the all-age anemia prevalence is 13.4%, and this value is 34% in western China (11, 12). In the Chinese population aged over 60 and above, the age-adjusted prevalence of anemia varies from 8.5 to 35.4% (13). In Asian people, anemia is negatively associated with handgrip strength, muscle mass, and physical performance (14–16), all of which are components in sarcopenia diagnosis.

Hemoglobin is the well-established way that clinical practitioners establish the diagnosis of anemia. As anemia is associated with sarcopenia, it can be assumed that a low level of hemoglobin may associate with sarcopenia (17). Insufficient hemoglobin can affect the oxygen delivery to skeletal muscle, and negatively impact muscle strength, as observed in people with chronic hypoxia (18). Anemia is also positively associated with multiple inflammatory markers, which may affect muscle mass and physical performance in a negative way (18). A few studies have proposed that hemoglobin was positively associated with muscle strength and physical function (17, 19). Even so, these studies have mostly been with small-size samples (17), cross-sectional design (20, 21), or applied sarcopenia diagnosis criteria which is unsuitable in Asian populations (22).

Alerted by the insufficient clinical awareness of sarcopenia, the large Chinese population vulnerable to anemia and low hemoglobin, and the possible mechanism between hemoglobin and sarcopenia components, the association between hemoglobin and sarcopenia in Chinese population should be thoroughly explored. To the best of our knowledge, no large-scale studies of the Chinese population have elucidated the association of hemoglobin level with sarcopenia and its components using guidelines tailored for the Chinese. Therefore, we used a nationally representative, population-based survey [the China Health and Retirement Longitudinal Study (CHARLS)] to explore the aforementioned associations, with the aim of bridging the knowledge gap.

## Methods

### Study population

The CHARLS is a national, population-based survey focusing on Chinese aged 45 years and above. A total of 450 representative communities from 28 provinces were selected using a multistep probability sampling strategy (23). The first survey was started in 2011, and participants were followed every 2 years. A total of 17,705 respondents were interviewed in 2011, 18,605 respondents were interviewed in 2013, and 21,095 people were interviewed in 2015. Because biomarker and blood tests were only conducted in 2011 and 2015, data from these years were used. The inclusion criteria were (1) individuals aged 60 years or above in 2011; (2) available data regarding sarcopenia status; and (3) the possession of blood test data in 2011. People missing demographic or health information were excluded.

This study contained two sub-studies. (1) A cross-sectional analysis of the CHARLS 2011 population. Of the total of 17,705 participants, 14,650 people were excluded because of missing blood test data (*n* = 8,293), no sarcopenia relevant data (*n* = 2,341), no demographic or health information (*n* = 808), and ages below 60 years (*n* = 3,208). A total of 3,055 participants remained for the analysis. (2) In the cohort analysis, we further excluded 831 people who had either possible sarcopenia or sarcopenia in 2011 and removed 1,202 participants who had no sarcopenia data in 2015; thus, constructed a cohort of 1,022 people.

The Institutional Review Board at Peking University approved CHARLS (approval number: IRB00001052-11014 for biomarker collection; IRB00001052-11015 for main household survey including anthropometrics), and all of the participants were required to provide written informed consent before joining CHARLS.

### Assessment of sarcopenia and its components

The first expert consensus on sarcopenia in Chinese population (5) was published in 2021. The Chinese consensus highly considered the guideline issued by the Asian Working Group for Sarcopenia (AWGS) and recommended the use of its cutoff values for sarcopenia diagnosis in Chinese population (24). Participants' sarcopenia status was assessed by three components: appendicular skeletal muscle mass (ASM), muscle strength, and physical performance.

The ASM was estimated using a validated equation derived from Chinese adults (25). The equation has been applied in research which has similar study populations as that of our study (26, 27). The equation is:


$$\begin{array}{l} ASM=0.193^{*}weight\left(inkg\right)+0.107^{*}height\left(incm\right)-\\ \;4.157^{*}sex\left(Male=1,Female=2\right)-0.037^{*}age\\ \;(inyear)-2.631 \end{array}$$

Participants' height and weight were measured using a stadiometer and a digital floor scale, respectively, to the nearest 0.1 cm and 0.1 kg. The ASM derived from the abovementioned equation is consistent with the result from dual X-ray absorptiometry (DXA) (25). In clinical practice, DXA requires specialized radiology equipment and experienced physicians to ensure testing accuracy. Bioelectrical impedance analysis (BIA) is a less expensive assessment technology and requires no specialists to perform. Both DXA and BIA are recommended for muscle mass evaluation, and their results are interchangeable (24, 28). BIA criteria were used in this study to make findings applicable in a more generalized setting. Low muscle mass was defined as a height-adjusted muscle mass (ASM/Height^2^) <7.0 kg/m^2^ for males and below 5.7 kg/m^2^ for females (5).

Muscle strength was measured *via* handgrip strength, which was evaluated by asking participants to hold the dynamometer at a right angle (90°) and squeeze a YuejianTM WL-1000 dynamometer (Nantong Yuejian Physical Measurement Instrument Co., Ltd., Nantong, China) two times in each hand as hard as possible. The maximum reading was used for the sarcopenia diagnosis. The cutoff points for low muscle strength were 28 kg for males and 18 kg for females. CHARLS used a 5-time chair stand test to evaluate physical performance. The cutoff value of low physical performance was a test time ≥ 12 s for all sexes (29).

Possible sarcopenia was defined as either low muscle strength or low physical performance without low muscle mass. Sarcopenia was diagnosed when low muscle mass plus either low muscle strength or low physical performance was identified. Severe sarcopenia was defined as the co-existence of low muscle mass, low muscle strength, and low physical performance. As only 166 (5.43%) participants had severe sarcopenia at baseline, severe sarcopenia was merged into sarcopenia to avoid sparse data bias (30). Participants were categorized into no sarcopenia (*n* = 1,618), possible sarcopenia (*n* = 726), and sarcopenia (*n* = 711).

### Blood sample collection and analysis

The CHARLS project collaborated with the Chinese Center for Disease Control and Prevention (China CDC) to collect and process blood samples. Three tubes of blood were collected from each participant. One tube was immediately stored at 4°C and transported to the nearest CDC center or health center for complete blood count; the median time from collection to analysis was 97 min. The other two tubes were stored at −80°C for bioassay analysis at a national certified lab at Capital Medical University (31). Cystatin C is a protein associated with muscle mass in some chronic disease patients (32). In the CHARLS project, it was measured using a particle-enhanced turbimetric assay with a detection range of 0.5–8.0 mg/L.

### Covariates

CHARLS participants were interviewed using a computer-aided structured questionnaire. Demographic information, such as age, sex, socioeconomic level, and urban/rural residence, was collected. Socioeconomic level was collected from the participants' self-evaluation scale. Health status and functioning data, including smoking, drinking, body mass index (BMI), blood pressure, and diagnoses of hypertension/dyslipidemia/diabetes/kidney disease/heart failure/rheumatism, were collected. A participant was identified as a patient with the abovementioned diseases if the participant had been diagnosed by physicians or was on medication at the time of interview. Hypertension was defined as systolic blood pressure ≥ 140 mmHg and/or diastolic pressure ≥ 90 mmHg or if the participant was on medications by the time of the interview. Diabetes was identified if the participant was on antidiabetic agents or had plasma glucose ≥ 200 mg/dl. BMI was categorized into underweight (below 18.5 kg/m^2^), normal weight (18.5–23.9 kg/m^2^), and overweight or obese (24 kg/m^2^ and above).

### Statistical analysis

Continuous data were presented as the mean with standard deviation (SD) or median with interquartile range (IQR). Categorical data are presented as *n* (%). The baseline data of all 6,263 participants were summarized and stratified by their sarcopenia status in the baseline year 2011. Comparisons of baseline characteristics among the groups were conducted using the Kruskal–Wallis test. Logistic regression was then performed to identify the association of hemoglobin with sarcopenia and with sarcopenia components (ASM, muscle strength, and physical performance).

All the abovementioned associations were then evaluated in a cohort analysis. As the sarcopenia test was conducted on the day of the interview, the follow-up period was defined as the interval between the interview day in 2011 and the interview day in 2015. Schoenfeld's residuals showed no violation of the proportional hazards assumption (*P* = 0.44). Cox proportional hazard models were used to calculate hazard ratios (HRs) with 95% confidence intervals (CIs). The sex-specific association was then explored. Finally, analyses were conducted in the following subgroups: residence, BMI level, drinking, and smoking. All the analyses were performed using STATA 16.0/MP (StataCorp, USA). A two-sided *P*-value < 0.05 was considered to be statistically significant.

## Results

### Baseline statistics of the study population

The baseline statistics of the study population are presented in Table 1, as stratified by sarcopenia status in the baseline year. In the total of 3,055 participants, the prevalence of sarcopenia was 23.27%, and the prevalence of possible sarcopenia was 23.76%. Sarcopenia was more common in people of higher age, females, rural residents, unmarried people, less educated people, low socioeconomic level people, and people with low BMI/arthritis/rheumatism. People with higher cystatin C and lower hemoglobin were more commonly found to have sarcopenia (Table 1).

**Table 1:** Baseline characteristics of 3,055 CHARLS participants, stratified by their sarcopenia status in year 2011.

**Characteristics**	**No sarcopenia**	**Possible sarcopenia**	**Sarcopenia**
Number of people, *n* (row %)	1,618 (52.96)	726 (23.76)	711 (23.27)
Age (year), mean (SD)	66.75 (5.48)	68.28 (6.10)	71.84 (6.81)
Male, *n* (%)	909 (56.18)	401 (55.23)	262 (36.85)
Urban residence, *n* (%)	575 (35.54)	270 (37.19)	191 (26.86)
Married, *n* (%)	1,360 (84.05)	597 (82.23)	504 (70.89)
Smoking, *n* (%)	743 (45.92)	335 (46.14)	253 (35.58)
Drinking, *n* (%)	583 (36.03)	199 (27.41)	177 (24.89)
**Educational level**, ***n*** **(%)**			
No formal education	482 (29.79)	263 (36.23)	398 (55.98)
Primary	788 (48.70)	349 (48.07)	268 (37.69)
Secondary	313 (19.34)	109 (15.01)	44 (6.19)
Tertiary and above	35 (2.16)	5 (0.69)	1 (0.14)
**Socioeconomic level**, ***n*** **(%)**			
Above average	50 (3.09)	25 (3.44)	20 (2.81)
Average	893 (55.19)	386 (53.17)	355 (49.93)
Relatively poor	501 (30.96)	219 (30.17)	210 (29.54)
Poor	174 (10.75)	96 (13.22)	126 (17.72)
BMI (kg/m^2^), mean (SD)	22.98 (3.70)	25.24 (3.40)	19.90 (2.19)
Systolic blood pressure (mmHg), mean (SD)	134.59 (22.11)	138.06 (22.33)	136.07 (25.07)
Diastolic blood pressure (mmHg), mean (SD)	74.85 (11.45)	76.27 (11.86)	73.02 (12.23)
**Comorbidities**, ***n*** **(%)**			
Hypertension	467 (28.86)	306 (42.15)	181 (25.46)
Dyslipidemia	164 (10.14)	98 (13.50)	46 (6.47)
Diabetes	99 (6.12)	68 (9.37)	26 (3.66)
Kidney disease	101 (6.24)	67 (9.23)	35 (4.92)
Heart disease	224 (13.84)	139 (19.15)	116 (16.32)
Arthritis or rheumatism	569 (35.17)	301 (41.46)	306 (43.04)
Cystatin C (mg/l), mean (SD)	1.08 (0.27)	1.11 (0.29)	1.16 (0.34)
Hemoglobin (g/dl), mean (SD)	14.34 (2.22)	14.64 (2.27)	13.58 (2.02)
**Sarcopenia components, mean (SD)**			
Height-adjusted ASM (kg/m^2^)	6.68 (1.13)	7.10 (0.86)	5.57 (0.91)
Handgrip strength (kg)	33.02 (8.34)	28.20 (9.07)	23.34 (7.68)
Five-time chair stand time (s)	9.16 (1.76)	15.42 (6.20)	14.97 (5.33)

% was shown as column percentage unless otherwise specified.

ASM, appendicular skeletal muscle mass; BMI, body mass index; SD, standard deviation.

### Cross-sectional analysis of the association of hemoglobin with sarcopenia and its components

After adjustments for demographic factors, a very significant association (*P* < 0.001) was found between hemoglobin and sarcopenia. This association was not significantly changed (*P* = 0.04) with further adjustments for health-related factors. On average, a per 1 g/dl higher hemoglobin level was associated with 5% lower odds of sarcopenia (OR = 0.95, 95% CI: 0.90–0.98) (Table 2).

**Table 2:** Cross-sectional associations among hemoglobin, sarcopenia, and sarcopenia components in baseline year 2011.

	**Model 1^a^**		**Model 2^b^**		**Model 3^c^**	
	**OR (95%CI)**	***P*-value**	**OR (95%CI)**	***P*-value**	**OR (95%CI)**	***P*-value**
**Sarcopenia**						
Hemoglobin	0.89 (0.85–0.93)	<0.001	0.94 (0.90–0.99)	0.02	0.95 (0.90–0.99)	0.04
**COMPONENT OF SARCOPENIA**						
**Low height-adjusted ASM**						
Hemoglobin	0.85 (0.82–0.89)	<0.001	0.90 (0.86–0.95)	<0.001	0.91 (0.86–0.97)	0.002
**Low muscle strength**						
Hemoglobin	0.94 (0.90–0.99)	0.02	0.96 (0.91–1.01)	0.08	0.96 (0.92–1.01)	0.16
**Low physical performance**						
Hemoglobin	1.03 (0.99–1.07)	0.11	1.02 (0.99–1.06)	0.19	1.02 (0.99–1.06)	0.22

ASM, appendicular skeletal muscle mass; CI, confidence interval; OR, odds ratio.

a Adjusted for sex, age, residence.

b Adjusted as Model 1 with further adjustments for marital status, socioeconomic level, education, smoking, drinking, body mass index.

c Adjusted as Model 2 with further adjustment for systolic blood pressure, diastolic blood pressure, hypertension, dyslipidemia, chronic kidney disease, heart disease, arthritis or rheumatism, cystatin C.

Among sarcopenia components, a statistical association was found between hemoglobin and low height-adjusted ASM. On average, a per 1 g/dl elevated hemoglobin level was associated with 9% lower odds of having low height-adjusted ASM (OR = 0.91, 95% CI: 0.86–0.97). It should be noted that although no statistical significance (*P* = 0.16) was found in the association between hemoglobin and muscle strength, the upper 95% CI is close to 1.00. No evidence (*P* = 0.22) between hemoglobin and low physical performance was found.

### Cohort analysis of the association of hemoglobin with sarcopenia and its components

A total of 1,022 people who had no sarcopenia in 2011 were followed up to 2015; 165 people (13.41%) were diagnosed with sarcopenia, with an incidence rate of 336.61 per 10,000 person-years.

After adjusting for multiple covariates, consistent evidence was found between baseline hemoglobin and sarcopenia (HR = 0.92, 95% CI: 0.84–1.00), low height-adjusted ASM (HR = 0.95, 95% CI: 0.90–1.00), and low physical performance (HR = 0.92, 95% CI: 0.85–0.99). No association between hemoglobin and low muscle strength was observed (Table 3).

**Table 3:** Longitudinal associations among baseline hemoglobin level, sarcopenia, and sarcopenia components, 2011–2015.

	**Model 1^a^**		**Model 2^b^**		**Model 3^c^**	
	**HR (95%CI)**	***P*-value**	**HR (95%CI)**	***P*-value**	**HR (95%CI)**	***P*-value**
**Sarcopenia**						
Hemoglobin	0.87 (0.79–0.95)	0.003	0.92 (0.84–1.01)	0.08	0.92 (0.84–1.00)	0.05
**COMPONENT OF SARCOPENIA**						
**Low Height-adjusted ASM**						
Hemoglobin	0.90 (0.85–0.95)	<0.001	0.94 (0.89–1.00)	0.04	0.95 (0.90–1.00)	0.05
**Low muscle strength**						
Hemoglobin	0.94 (0.87–1.03)	0.17	0.97 (0.89–1.06)	0.52	0.98 (0.90–1.06)	0.60
**Low physical performance**						
Hemoglobin	0.92 (0.83–0.99)	0.04	0.92 (0.85–0.99)	0.03	0.92 (0.85–0.99)	0.03

ASM, appendicular skeletal muscle mass; CI, confidence interval; HR, hazard ratio.

a Adjusted for sex, age, residence.

b Adjusted as Model 1 with further adjustments for marital status, socioeconomic level, education, smoking, drinking, body mass index.

c Adjusted as Model 2 with further adjustment for systolic blood pressure, diastolic blood pressure, hypertension, dyslipidemia, chronic kidney disease, heart disease, arthritis or rheumatism, cystatin C.

Due to the difference in the cutoff value of sarcopenia criteria between sexes, the sex-specific association between baseline hemoglobin and sarcopenia was explored. In males, hemoglobin was associated with sarcopenia, and a per 1 g/dl increase in hemoglobin was associated with a 10% lower rate of sarcopenia (HR = 0.90, 95% CI: 0.83–0.99). Hemoglobin in males was also statistically associated with low height-adjusted ASM (HR = 0.92, 95% CI: 0.89–0.96) and with low physical performance (HR = 0.91, 95% CI: 0.85–0.97). In females, hemoglobin was found to be marginally associated with sarcopenia (HR = 0.92, 95% CI: 0.84–1.00) and with low height-adjusted ASM (HR = 0.96, 95% CI: 0.93–1.00); strong evidence of association was also found in low physical performance (HR = 0.92, 95% CI: 0.86–0.99). Hemoglobin was not evidently associated with low muscle strength in all sexes (Supplementary Table S1).

### Subgroup analysis for baseline hemoglobin and its association with sarcopenia

**Figure 1:**
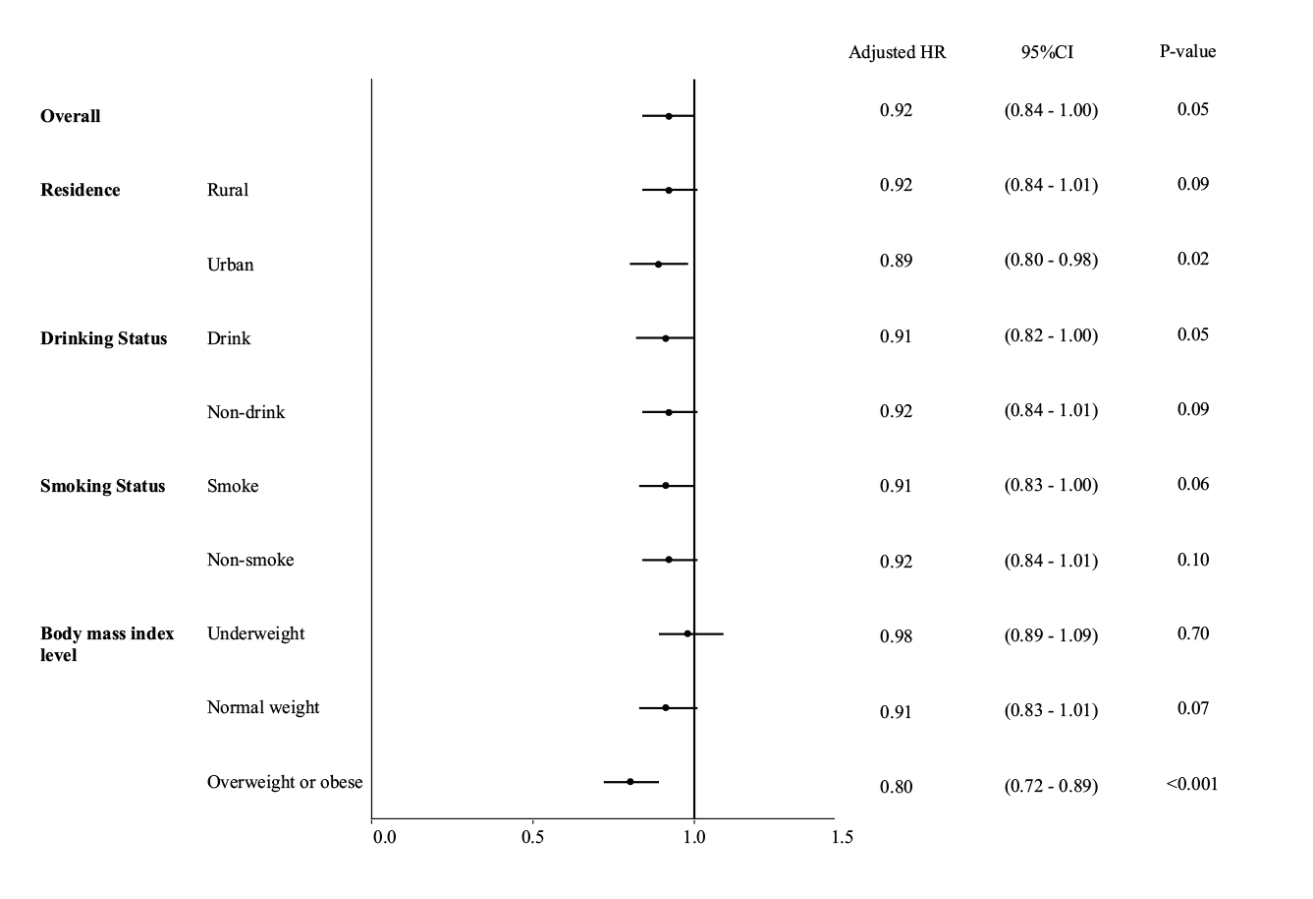
Forest plot for multivariable Cox regression results to show the sex-specific association between hemoglobin and sarcopenia, stratified by residence, body mass index level, drinking status, and smoking status.

Figure 1 showed that the association between hemoglobin and sarcopenia was stronger in urban residents (HR = 0.89, 95% CI: 0.80–0.98). Among people of different BMI levels, the hemoglobin-sarcopenia association was stronger in people with higher BMI levels. In the overweight/obese group, a per 1 g/dl higher hemoglobin was associated with a 20% lower hazard rate of sarcopenia (HR = 0.80, 95% CI: 0.72–0.89), with very strong evidence (*P* < 0.001). No evidence was found in underweight people. The hemoglobin-sarcopenia association was similar in people with various drinking and smoking statuses (Figure 1).

## Discussion

In this study, the cross-sectional analysis demonstrated hemoglobin in Chinese individuals aged 60 and above was negatively associated with sarcopenia and low height-adjusted ASM. The cohort study showed that in the general population, hemoglobin was negatively associated with sarcopenia, low muscle mass, and low physical performance. In both sexes, hemoglobin was negatively associated with sarcopenia, low muscle mass, and physical performance, although the magnitude of association varies. No evidence of the association between hemoglobin and muscle strength was found. Subgroup analyses demonstrated that urban residents and people with higher BMI levels had a large magnitude of hemoglobin-sarcopenia association.

The overall prevalence of sarcopenia in the study population was 13.41%, with a higher prevalence in females, similar to other studies on Chinese population (33, 34). Our findings were largely consistent with several previous studies (18, 21). However, there were still some discrepancies in this field. A cross-sectional study on a Taiwanese population demonstrated that hemoglobin was only associated with physical performance and muscle strength, not with sarcopenia or muscle mass (17). Erythropoietin receptors are expressed in human skeletal muscle (35). Skeletal muscle mass is independently associated with body responsiveness to erythropoietin stimulating agents (36). It is fairly possible that insufficient skeletal muscle led to decreased hemoglobin production, caused a reverse causality that could not be ruled out in a cross-sectional design and biased the result in that Taiwanese study.

There is a paucity of longitudinal research on hemoglobin and its association with sarcopenia, and there is even more scarce research on sex-specific associations. Along with aging, the decline in serum testosterone in males is associated with compromised hematopoiesis, further inhibiting hemoglobin production (37–39). The decline in hemoglobin in men usually starts in their 30s, whereas hemoglobin in women is slightly increased after menopause before starting to decline in their 60s (40). In the study population, the hemoglobin level in the oldest quartile of males (mean age: 77.5 years) was on average 92.5% of that in the youngest quartile (mean age: 61.5 years); in females, the mean hemoglobin in the oldest quartile (mean age: 77.4 years) was 96.4% of that in the youngest quartile (mean age: 61 years). The above relatively smaller decline in hemoglobin in females as compared to males may explain the sex-specific differences in the magnitude of the association of hemoglobin with sarcopenia, ASM, and physical performance.

This study did not find evidence of the association of hemoglobin with muscle strength in either the cross-sectional or cohort analyses. This finding was consistent with a study on older individuals (41). Yet, another study demonstrated hemoglobin was associated with handgrip strength in older individuals (42). It is possible the higher average age of the participants (over 75 years) and the different sample selections of the above study may justify the conflicting results.

In the cohort study, we found a consistent association of hemoglobin with physical performance. The current research on the association between hemoglobin and physical performance remains controversial. Studies using Functional Independence Measure (43) demonstrated the association of hemoglobin with physical function and performance. A systematic review of fifteen randomized clinical trials and five observational studies reported a negative association between hemoglobin and fatigue but not with physical function (44). We solely evaluated physical performance by the 5-time chair stand test without the gait speed test, and thus the evaluation may not fully reflect the participants' actual physical performance. The CHARLS gait speed test was set at a 2.5 m distance, which was much shorter than the recommended six meters (24). The validity of the 2.5 m distance is unclear, although the 3-meter, 6-meter, and longer distance gait speed tests have been validated (45, 46). As participants were more capable of completing a shorter distance, the 2.5 m test may likely overestimate the gait speed. Only 41.90% of people had both chair stand test and gait speed test records. Statistically significant differences in height, weight, and handgrip strength were found between individuals with gait speed records and those without. Using people with available gait speed in this study will heavily limit the sample size and may bias the study result.

Urban residents and obese/overweight people had a larger magnitude of hemoglobin-sarcopenia association; the underlying mechanism accounting for the larger magnitude remains unclear. Urban residents are known to have higher hemoglobin levels than rural residents (47), and a higher BMI level has an inverse association with hemoglobin in older Chinese people (48). It may be possible that there is a threshold, and only an over-threshold hemoglobin level is associated with sarcopenia. An in-depth analysis of urban/rural residents and people of different BMI groups is needed to further explore the abovementioned findings.

From a perspective of primary prevention and public health, increasing hemoglobin levels in people aged 60 and above can be better achieved through a multidisciplinary approach. Providing access to nutrient-dense foods that are rich in iron, vitamin B12, and folic acid. Older adults may have a reduced appetite or difficulty chewing, so it is important to provide meals that are easy to prepare, chew, and digest. In addition to dietary changes, regular exercise can help increase hemoglobin levels while strengthening muscle and improving physical performance. Low-impact aerobic activities such as walking, swimming, and biking may help increase circulation and promote healthy oxygen levels in the body. Moderate strength training can also help increase hemoglobin levels and build muscle strength to prevent sarcopenia (49). Excessive alcohol intake (over 2 alcoholic drinks/day) should be restricted as they can lead to a decrease in hemoglobin levels (50). It should consider the unique needs and challenges older adults face when designing interventions and involve healthcare providers, community organizations, and family caregivers in the process.

Despite all the efforts made in this study, there were several limitations. First, the core element of our research, the ASM, was calculated using a validated formula instead of BIA/DXA methods. Admittedly, using the formula may impair the generalization of our findings in non-Chinese population. However, this is unavoidable because our study was a secondary research using existing data. Considering the scale and representativeness of CHARLS, along with the consistence in our findings, we believe that this study makes its contribution to solving the urgent public health issue brought by sarcopenia. Readers are advised to be aware of the possible bias of ASM evaluation. Second, gait speed is an indicator of the overall health of the elderly (51). Without adjusting gait speed, our findings may be biased toward the null. Third, confounding factors, such as inflammatory biomarkers and dietary patterns, were not adjusted due to data availability. All this can confound the results. Finally, selection bias, including volunteer bias, should be considered when interpreting our results.

To conclude, our study demonstrated that hemoglobin level is negatively associated with sarcopenia in the Chinese population aged 60 and above. Males, urban residents, and people with a high BMI have a larger magnitude of the negative association between hemoglobin level and sarcopenia. Along with the high prevalence of sarcopenia and an aging society, our findings may generate meaningful implications for preventing sarcopenia and promoting healthy aging in China.

## Data availability statement

Publicly available datasets were analyzed in this study. This data can be found here: https://charls.pku.edu.cn/.

## Ethics statement

All procedures performed in studies involving human participants were in accordance with the ethical standards of the institutional and/or national research committee and with the 1964 Helsinki declaration and its later amendments or comparable ethical standards. The Institutional Review Board at Peking University approved CHARLS (approval number: IRB00001052-11014 for biomarker collection; IRB00001052-11015 for main household survey including anthropometrics), and all of the participants were required to provide written informed consent before joining CHARLS. Data were fully anonymized and it is impossible to re-identify any participants.

## Author contributions

QL and CH conceived the protocol. QL, JY, MZ, ZW, and YG contributed to the analysis and interpretation of data. QL and JY drafted the manuscript. QL critically revised the manuscript. All authors agree to be fully accountable for ensuring the integrity and accuracy of the work and read and approved the final manuscript.
