# Supplementary material for: Hemoglobin level is negatively associated with sarcopenia and its components in Chinese aged 60 and above
Source: Front Public Health. 2023 Mar 13;11:1081843. doi: 10.3389/fpubh.2023.1081843 (PMC10040688; doi:10.3389/fpubh.2023.1081843)
Supplement: Supplementary file 1 [file Table_1.DOCX]

Table S1: Sex-specific longitudinal associations between baseline hemoglobin level and sarcopenia, 2011 - 2015

|  | Male**^a^** | |  | Female**^a^** | |
| --- | --- | --- | --- | --- | --- |
|  | HR (95%CI) | P-value |  | HR (95%CI) | P-value |
| Sarcopenia |  |  |  |  |  |
| Hemoglobin | 0.90 (0.83 - 0.99) | 0.03 |  | 0.92 (0.84 - 1.00) | 0.03 |
| Component of sarcopenia |  |  |  |  |  |
| Low Height-adjusted ASM |  |  |  |  |  |
| Hemoglobin | 0.92 (0.89 - 0.96) | <0.001 |  | 0.96 (0.93 - 1.00) | 0.08 |
| Low muscle strength |  |  |  |  |  |
| Hemoglobin | 0.96 (0.86 - 1.08) | 0.51 |  | 0.96 (0.84 - 1.10) | 0.58 |
| Low physical performance |  |  |  |  |  |
| Hemoglobin | 0.91 (0.85 - 0.97) | 0.004 |  | 0.92 (0.86 - 0.99) | 0.02 |

ASM appendicular skeletal muscle mass; CI confidence interval; HR hazard ratio

**^a^**Adjusted for age, residence, marital status, socioeconomic level, education, smoking, drinking, body mass index, systolic blood pressure, diastolic blood pressure, hypertension, dyslipidemia, chronic kidney disease, heart disease, arthritis or rheumatism, cystatin C
